# SNAPPE II: ANALYSIS OF ACCURACY AND DETERMINATION OF THE CUTOFF POINT AS A DEATH PREDICTOR IN A BRAZILIAN NEONATAL INTENSIVE CARE UNIT

**DOI:** 10.1590/1984-0462/2020/38/2019029

**Published:** 2020-12-16

**Authors:** Maria Marcia Farias Trajano Fontenele, Cristiana Ferreira Silva, Álvaro Jorge Madeiro Leite, Eveline Campos Monteiro Castro, Francisco Herlânio Costa Carvalho, Ana Valeska Siebra e Silva

**Affiliations:** a Universidade Federal do Ceará, Fortaleza, CE, Brazil.

**Keywords:** Infant mortality, Neonatal mortality, Severity of illness index, Mortalidade infantil, Mortalidade neonatal, Índice de gravidade de doença

## Abstract

**Objective:**

To analyze the accuracy of the Score for Neonatal Acute Physiology Perinatal Extension (SNAPPE II) as a death predictor, to determine the cutoff point for mortality, and to analyze the association of independent variables with death.

**Methods:**

Prospective, longitudinal, hospital-based study on newborns admitted to the Neonatal Intensive Care Unit (NICU) for the first time from November 1, 2016 to April 30, 2017. Newborns with less than 12 hours of length of stay at the NICU, out-of-hospital births, major congenital malformations, and inter-hospital transfer were excluded. Variables were grouped according to hierarchical framework, related to maternal characteristics (distal level), prenatal and childbirth care (intermediate level), and birth conditions (proximal level). Descriptive analyses of SNAPPE II score ranges, Receiver Operating Characteristics Curve (ROC curve) to define the cutoff point for mortality, and bivariate analysis by the Wald test and multiple logistic regression were conducted.

**Results:**

After selection, the sample consisted of 247 newborns. In this study, the SNAPPE II cutoff point for mortality was 27, with sensitivity of 84.1% and specificity of 82.4%. 61% of those with a score ≥27 died. Multiple logistic regression showed an association between death and proximal-level variables: sepsis (Odds Ratio [OR] 10.68; 95% confidence interval [95%CI] 2.82–40.48; p<0.001); SNAPPE II ≥27 (OR 5.85; 95%CI 1.90–18.05; p=0.002); birth weight 750–999 g (OR 4.15; 95%CI 1.06–16.14; p=0.040); and nonuse of surfactant (OR 0.159; 95%CI 0.04–0.53; p=0.003).

**Conclusions:**

Neonatal mortality was directly proportional to increase in SNAPPE II. Score≥27 increased the odds of dying by six times compared with neonates with lower scores. The proximal variables related to health conditions and neonatal care were associated with death.

## RESUMO

**Objetivo:**

Analisar a acurácia do *Score for Neonatal Acute Physiology Perinatal Extension* (SNAPPE II) como preditor de óbito, determinar o ponto de corte para mortalidade e analisar a associação das variáveis independentes com óbito.

**Métodos:**

Estudo prospectivo, longitudinal, de base hospitalar com recém-nascidos admitidos pela primeira vez na Unidade de Terapia Intensiva Neonatal (UTIN) entre 1° de novembro de 2016 e 30 de abril de 2017. Foram excluídos recém-nascidos com permanência menor que 12 horas na UTIN, nascimento extra-hospitalar, malformações congênitas maiores e transferência inter-hospitalar. As variáveis foram agrupadas sob determinação hierarquizada, relacionadas a características maternas (nível distal), assistência ao pré-natal e parto (nível intermediário) e condições do nascimento (nível proximal). Foram conduzidas análises descritivas dos graus de pontuação do SNAPPE II, *Receiver Operating Characteristics Curve* (curva ROC) para definição do ponto de corte para mortalidade e análise bivariada pelo teste de Wald e regressão logística múltipla.

**Resultados:**

Após seleção, a amostra constituiu-se de 247 recém-nascidos. Neste estudo, o ponto de corte do SNAPPE II para mortalidade foi 27, com sensibilidade de 84,1% e especificidade de 82,4%. Evoluíram a óbito 61% daqueles com pontuação ≥27. A regressão logística múltipla mostrou associação entre óbito e variáveis de nível proximal: sepse (*Odds Ratio* [OR] 10,68; intervalo de confiança de 95% [IC95%] 2,82–40,48; p<0,001); pontuação ≥27 (OR 5,85; IC95% 1,90–18,05; p=0,002); peso ao nascer entre 750 e 999 g (OR 4,15; IC95% 1,06–16,14; p=0,040); e não uso de surfactante (OR 0,159; IC95% 0,04–0,53; p=0,003).

**Conclusões:**

A mortalidade neonatal foi diretamente proporcional ao aumento do SNAPPE II. Escore≥27 aumentou seis vezes a chance de óbito em relação aos neonatos com escore inferior. As variáveis proximais relacionadas às condições de saúde e da atenção neonatal associaram-se ao óbito.

## INTRODUCTION

In Brazil, neonatal mortality represents almost 70% of deaths in the first year of life, with predominance of the early neonatal component. Of these deaths, approximately 25% occur in the first 24 hours.^1^ ^,^ ^2^ About 60% of neonatal deaths in the country occur due to preventable causes, especially the adequate care for pregnant women, childbirth, and the newborn.^1^ ^,^ ^3^ The high rates of preventable perinatal and neonatal mortality in the least developed regions of Brazil can be addressed with the organization and management of care at different levels of health care.^4^

The use of indicators, scales, and other instruments enables the management to assess the provision of care. In neonatal units, the comparison of mortality rates between services is an excellent quality indicator. Thus, scores were developed to identify the degree of severity, enabling the systematization and adequacy of care, with subsequent reduction in mortality.^5^

In 2001, Richardson and collaborators developed the *Score for Neonatal Acute Physiology Perinatal Extension* (SNAPPE II) instrument for assessing the newborns' clinical severity in the Neonatal Intensive Care Unit (NICU). This index is easy to apply, can be used in all neonates, regardless of birth weight and gestational age, and proved to be a good predictor of mortality. It is based on multiple physiological changes: blood pressure, temperature, urine output, serum pH, PaO_2_/FiO_2_ ratio, and the presence of multiple seizures, pinpointing the worst moments in the first 12 hours of admission. It also assesses perinatal factors: birth weight, classification of small for gestational age (SGA) below the third percentile (<P3), and Apgar score in the 5^th^ minute <7. The higher the score, the greater the risk of death.

The SNAPPE II cutoff point related to death must be individualized for each service,^6^ making it necessary to constitute a management practice. In the maternity ward where this study was conducted, there were no studies that showed the clinical severity of newborns. Therefore, this research aimed to analyze the accuracy of the SNAPPE II severity score as a predictor of death at the NICU, in a tertiary maternity hospital in the city of Fortaleza (state of Ceará, Brazil), and to determine the predictive cutoff point for mortality. In addition, it intends to relate the score to the period of death and to analyze its association with variables.

## METHOD

Prospective, longitudinal, hospital-based study, conducted in the NICUs of the Maternidade Escola Assis Chateaubriand (MEAC), at Universidade Federal do Ceará (UFC), of tertiary level, linked to the public network and a reference for obstetric and neonatal care. The maternity hospital serves, on average, 500 births per month, 700 premature babies per year, and has two NICUs with 21 beds.

For the sample calculation, the reference for finite population was used, considering data about the maternity from 2015, with a population of 565 neonates admitted to the NICU, 26% prevalence of mortality, and 5% sampling error, totaling 200 newborns. Data on neonates admitted to the NICUs in a six-month period were collected: newborns with a ≥12-hour length of hospital stay at the first admission to NICU were included; newborns with major congenital malformations, those born outside the study hospital environment, and those transferred to other hospitals were excluded. Data were collected from medical records with the completion of questionnaires with maternal and neonatal variables and variables related to SNAPPE II.

The dependent variable was death in the NICU. The independent variables were organized based on an adaptation of the hierarchical framework for investigating neonatal infant death,^7^ grouped into three hierarchical groups and organized at intermediate I, II, and proximal levels in relation to the outcome. The considered variables were related to the newborn's, perinatal, and care conditions: intermediate level I (group I) – maternal characteristic and morbidity: maternal age, gestational hypertension, and multiple pregnancy; intermediate level II (group II) – prenatal and childbirth care: prenatal care,^8^ use of antenatal corticosteroids (at least one dose administered), type of childbirth; proximal level (group III) – sex, health conditions of the newborn, and neonatal care: gestational age – calculated by the best obstetric estimate or, in its absence, by the neonatal physical examination –,^9^ birth weight: categories used in SNAPPE II (<750, 750–999, >999 g); Apgar score in the 1^st^ and 5^th^ minutes of life; resuscitation in the delivery room (positive pressure ventilation carried out with a balloon and tracheal mask or cannula, associated or not with cardiac massage and/or the use of medications); respiratory distress syndrome (RDS); use of surfactant; pneumothorax; patent ductus arteriosus (PDA), defined according to clinical changes and echocardiographic confirmation;^10^ peri-intraventricular hemorrhage (PIVH) grade III or IV, according to Papile et al.;^11^ sepsis confirmed with blood culture or cerebrospinal fluid (CSF) culture; necrotizing enterocolitis (NEC) stages II or III;^12^ SNAPPE II, categorized based on its cutoff point for mortality; variables included in the score (to assess the strength of association of the isolated variables, highlighting which ones would be more intense for determining the outcome): mean arterial pressure: <20, 20–29, and ≥30 mmHg; armpit temperature in the first 12 h: <35; 35–35.5, and> 35.5°C; PaO_2_/FiO_2_ ratio: <0.3, 0.3–0.99, and 1.00–2.49; pH: <7.10, 7.10–7.19, and >7.19; urine output: <0.1, 0.1–0.9 mL/kg/h; multiple seizure (more than one seizure episode in the first 12 hours of admission to the NICU); SGA <P3. The lowest PaO_2_/FiO_2_ ratio and the lowest pH were obtained from arterial blood gas test in the first 12 hours of admission of patients in assisted ventilation (mechanical ventilation and Continuous Positive Airway Pressure – CPAP); for those without ventilator support, a score of zero was attributed, following the recommendation of the SNAPPE II authors.

The *Statistical Package for the Social Sciences* (SPSS) 18.0 program was used for statistical analysis. The Odds Ratio (OR) was employed to assess the relationship between independent and dependent variables, with a 95% confidence interval (95%CI).

A descriptive analysis of SNAPPE II was performed in the total population and in relation to death and discharge from the NICU, with measures of central tendency (mean and median) and dispersion (standard deviation) by the Mann-Whitney U test, in addition to absolute and relative frequencies of the SNAPPE II score ranges of the total population and regarding death and discharge from the NICU. Birth weight, gestational age, and SNAPPE II were correlated by the Spearman's Rho coefficient. The Receiver Operating Characteristics Curve (ROC curve) was constructed to obtain the cutoff point of SNAPPE II for mortality. The cutoff point with the highest value of the ordinate axis and the lowest value of the abscissa axis was selected as the cutoff point for mortality. The discriminatory performance of the curve was verified by calculating the area under the curve (AUC).

Bivariate analysis was performed with the grouping of independent variables according to the hierarchization presented in the conceptual model, frequency distribution, calculations of the gross OR and the statistical significance of association between independent variables and the dependent variable, and its respective 95%CI by the Wald test. Multiple logistic regression using the Stepwise Forward model (conditional) was tested with the variables from the previous step, considering p≤0.20, according to the hierarchization presented in the conceptual model, and the OR measure was used. The statistical significance of the associations was equal to 0.05 (5%), with 95%CI. The error component was measured by the Goodness of Fit test, with the calculation of the Hosmer-Lemeshow chi-square, and its p-value. The Nagelkerke's R^2^ coefficient of determination was calculated.

The standards for research involving human beings were respected according to Resolution No. 466/12 of the National Health Council, Brazilian Ministry of Health. The project was sent to Plataforma Brasil, submitted and approved by the MEAC Research Ethics Committee, under Opinion No. 1,783,207.

## RESULTS

During the collection period – from November 1, 2016 to April 30, 2017 – there were 2,471 births. A total of 383 neonates were admitted to the NICU; of these, 8 remained for less than 12 hours; 2 were born outside the institution; 34 had major congenital malformations; and 92 were transferred (10 due to congenital heart diseases and 82 due to overcrowding in the NICU; SNAPPE II mean of those transferred: 19±10), thus totaling a sample of 247 neonates. Patients were followed up until the outcome of the last case on June 21, 2017.

Of the 247 newborns, 25.5% (63) died and 74.5% (184) were discharged from the NICU. Among the deaths, 81% (51) occurred in the neonatal period, with 58.8% (37) in the early neonatal period; of these, 18.9% (7) occurred in the first 24 hours; 22.2% (14), in the late neonatal period; and 19% (12), in the postneonatal period.

The distribution of live births included in the study regarding gestational age was: 19% (n=47) <28 weeks; 17% (n=42) between 28 and 31^6/7^ weeks; 19% (n=47) between 32 and 33^6/7^ weeks; 28.3% (n=70) between 34 and 36^6/7^ weeks; and 16.6% (n=41) ≥37 weeks. Of these patients, respectively, 85.1% (n=40), 28.6% (n=12), 6.4% (n=3), 7.1% (n=5), and 7.3% (n=3) died. Regarding birth weight of those included in the study, 19% (n=47) accounted for extremely low weight (<1000 g), with five <500 g; 14.2% (n=35), between 1000 and 1499 g; 38.5% (n=95), from 1500 to 2499 g; and 28.3% (n=70), ≥2500 g. Deaths according to weight ranges were 87.2% (n=41), 34.3% (n=12), 7.4% (n=7), and 4.3% (n=3), respectively.

The SNAPPE II mean of the total population was 27±21 and the median, 20, with minimum and maximum values of 0 and 110, respectively. Among deaths, the mean was 51±24 and the median, 47, and in those who were discharged, the values were 19±12 and 18, respectively.

**Graph 1:**
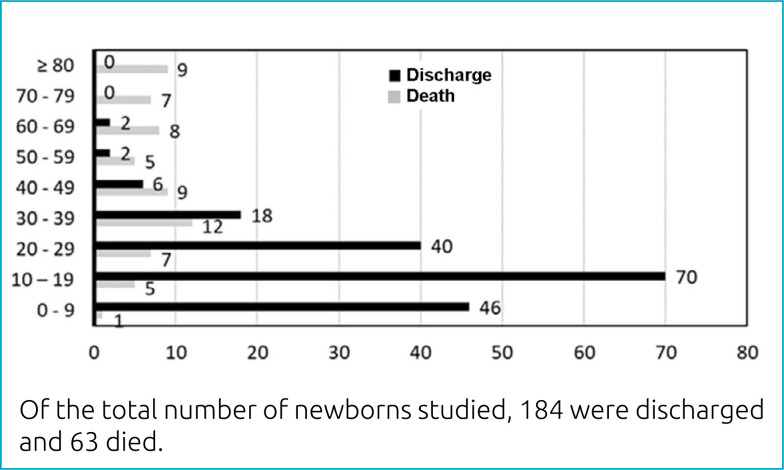
Newborns' severity profile and their respective outcomes in relation to the score ranges of the Score for Neonatal Acute Physiology Perinatal Extension.

The severity profile of newborns in the sample, in relation to the SNAPPE II score ranges and their outcomes, is described in Graph 1. In the analysis of the SNAPPE II score in relation to death in the NICU according to the Wald test, it was observed that each additional point in the score increases the odds of dying by 10% (OR 1.11; 95%CI 1.08–1.14; p<0.001).

**Figure 1:**
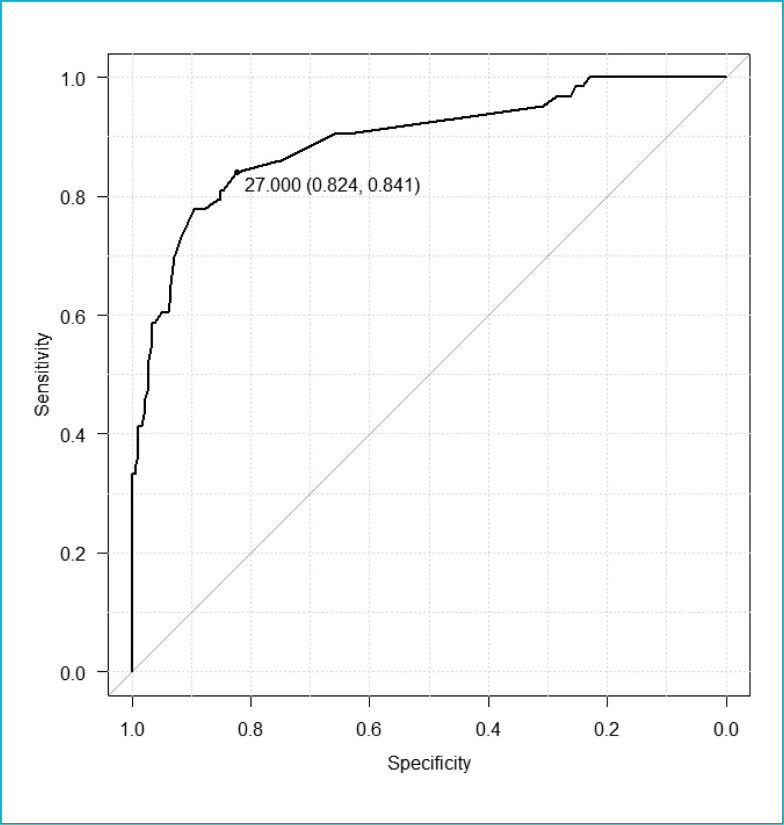
Receiver Operating Characteristics Curve for obtaining the cutoff point for mortality of the Score for Neonatal Acute Physiology Perinatal Extension.

The SNAPPE II mortality cutoff point was calculated by constructing the ROC curve with sensitivity of 0.84 and specificity of 0.82. The point found was 27, the AUC was 0.89, and the 95%CI, 0.84–0.94 (Figure 1).

The SNAPPE II score ranges (p=0.119) or its cutoff point for mortality (p=0.721) were not associated with the periods of occurrence of deaths – early neonatal, late neonatal, and postneonatal – according to the Fisher's test.

Correlations found from the Spearman's Rho coefficient between SNAPPE II and birth weight, and SNAPPE II and gestational age were: −0.525 and −0.518, respectively (p<0.0001); and, between gestational age and birth weight: 0.898 (p<0.0001).

The bivariate analysis between independent variables grouped in the proposed hierarchical framework and death is described in Tables 1, 2, and 3. The association of SNAPPE II≥27 with death is highlighted (OR 23.4; 95%CI 0.8–50.6; p <0.0001). Multiple logistic regression was conducted using the Stepwise Forward (conditional) model and tested with the variables from the previous step considering p≤0.20. Thus, according to tests of the method selection, the following variables were inserted: birth weight <750 and 750–999 g; sepsis; SNAPPEI≥27; nonuse of surfactant; PDA; NEC; gestational age <28, 28–31^6/7^ and 32–36^6/7^ weeks; and multiple pregnancy. However, in this model, the PDA variable showed an exacerbated variability in the confidence interval (95%CI 11.91–6,071.25) and a strong interaction effect on the other variables, in such a way the authors decided to remove it from the analysis. Therefore, the variables inserted in the model using the inclusion criterion of the method were: birth weight (categories <750 and 750–999 g), sepsis, SNAPPE II≥27, and nonuse of surfactant. After adjusting the variables, these remained associated with the outcome, except weight <750 g, thus composing the final model (Table 4). There was no collinearity between variables.

**Table 1:** Results of the bivariate analysis of maternal characteristics and morbidities, prenatal care, childbirth care, and neonatal demographics.

		Death in NICU	%	Survivor	%	Unadjusted OR	95%CI	p-value^a^
Maternal age (years)								
	<20	8	21.1	30	78.9	1.44	0.61–3.38	0.400
	≥35	10	21.3	37	78.7	1.01	0.35–2.88	0.980
	20–34	45	27.8	117	72.2	1.00		
Gestational hypertension								
	Yes	18	21.4	66	78.6	0.71	0.38–1.33	0.292
	No	45	27.6	118	72.4	1.00		
Multiple pregnancy								
	Yes	20	50.0	20	50.0	3,81	1.88–7.72	<0.001
	No	43	20.8	164	79.2	1.00		
Type of childbirth								
	Cesarean section	36	21.6	131	78.4	0.53	0.29–0.97	0.041
	Vaginal birth	27	33.8	53	66.2	1.00		
Prenatal care^b^								
	No	11	25.0	33	75.0	0.96	0.45–2.05	0.932
	Yes	52	25.6	151	74.4	1.00		
Antenatal corticosteroids^c^ (n=206)								
	No	14	31.8	30	68.2	1.17	0.57–2.41	0.658
	Yes	46	28.4	116	71.6	1.00		
Sex								
	Male	38	28.1	97	71.9	1.36	0.76–2.43	0.290
	Female	25	22.3	87	77.7	1.00		

NICU: Neonatal Intensive Care Unit; OR: Odds Ratio; 95%CI: confidence interval;

a Wald test;

b prenatal care adjusted for gestational age;

c neonates who were born before 37 weeks (n=206).

**Table 2:** Results of the bivariate analysis of the health conditions of the newborn and neonatal care.

		Death in NICU	%	Survivor	%	Unadjusted OR	95%CI	p-value^a^
GA (weeks)								
	<28 w	40	85.1	7	14.9	72.38	17.43–300.50	<0.001
	28–31 w 6 d	12	28.6	30	71.4	5.06	1.31–19.59	0.019
	32–36 w 6 d	8	6.8	109	93.2	0.93	0.23–3.68	0.917
	≥37 w	3	7.3	38	92.7	1.00		
Resuscitation								
	Yes	52	50.0	52	50.0	12.00	5.81–24.78	<0.001
	No	11	7.7	132	92.3	1.00		
RDS^c^ (n=206)								
	Yes	60	34.9	112	65.1	865433586.42	0.000	0.998
	No	0	0.0	34	100.0	1.00		
Apgar 1^st^ min^d^ (n=246)								
	<7	48	47.1	54	52.9	7.64	3.94– 14.80	<0.001
	≥7	15	10.4	129	89.6	1.00		
Use of surfactant^e^ (n=172)								
	No	7	7.9	82	92.1	0.048	0.02–0.12	<0.001
	Yes	53	63.9	30	36.1	1.00		
PDA^c^ (n=206)								
	Yes	14	48.3	15	51.7	2.65	1.19–5.92	0.010
	No	46	26.0	131	74.0	1.00		
PIVH grades III and IV (n=77)^f^								
	Yes	4	100.0	0	0.0	3744964458.33	0.000	0.999
	No	22	30.1	51	69.9	1.00		
Pneumothorax								
	Yes	8	72.7	3	27.3	8.77	2.25–34.21	0.002
	No	55	23.3	181	76.7	1.00		
NEC								
	Yes	13	72.2	5	27.8	9.30	3.16–27.35	<0.001
	No	50	21.8	179	78.2	1.00		
Sepsis^d^ (n=246)								
	Yes	16	66.7	8	33.3	7.65	3.08–18.98	<0.001
	No	46	20.7	176	79.3	1.00		

NICU: Neonatal Intensive Care Unit; 95%CI: confidence interval; OR: Odds Ratio;

a Wald test; GA: gestational age; RDS: respiratory distress syndrome; PDA: patent ductus arteriosus; PIVH: peri-intraventricular hemorrhage; NEC: necrotizing enterocolitis;

c 206 premature infants <37 weeks;

d 246 newborns, 1 unknown;

e 172 preterm infants with RDS;

f sample of 77 newborns (141 neonates ≥34 weeks and/or ≥500 g, 64 unknown).

**Table 3:** Bivariate analysis of constituent variables of the Score for Neonatal Acute Physiology Perinatal Extension.

		Death in NICU	%	Survivor	%	Unadjusted OR	95%CI	p-value^a^
MAP (mmHg)^b^ (n=246)								
	<20	15	88.2	02	11.8	38.90	8.48–178.45	0.999
	20–29	15	48.4	16	51.6	4.86	2.18–10.81	<0.001
	≥30	32	16.2	166	83.8	1.00		
Temperature (°C)								
	<35	24	82.8	5	17.2	30.88	10.71–89.05	<0.001
	35–35.5	16	34.0	31	66.0	3.32	1.57–7.00	0.002
	>35.5	23	13.5	148	86.5	1.00		
PaO_2_/FiO_2_ ratio (n=172)^c^								
	<0.3–0.99	11	50.0	11	50.0	2.06	0.83–5.08	0.116
	1–2.49	49	32.7	101	67.3	1.00		
pH (n=172)^g^								
	<7.1–7.19	12	80.0	3	20.0	9.08	2.45–33.66	0.001
	>7.19	48	30.6	109	69.4	1.00		
Urine output								
	<0.1	50	29.2	121	70.8	2.00	1.01–3.96	0.046
	0.1–0.9	13	17.1	63	82.9	1.00		
Birth weight (g)								
	<750	22	95.7	1	4.3	178.00	22.86–1,385.98	<0.001
	750–999	19	79.2	5	20.8	30.74	10.44–90.54	<0.001
	>999 g	22	11.0	178	89.0	1.00		
SGA<3^rd^ percentile								
	Yes	6	85.7	1	14.3	19.26	2.27–163.36	0.007
	No	57	23.8	183	76.2	1.00		
Apgar 5^th^ min								
	<7	21	77.8	6	22.2	14.83	5.63–39.03	<0.001
	≥7	42	19.1	178	80.9	1.00		

NICU: Neonatal Intensive Care Unit; 95%CI confidence interval; OR: Odds Ratio;

a Wald test; MAP: mean arterial pressure;

b sample of 246 newborns, 1 unknown;

c newborns in assisted ventilation whose arterial blood gas was collected in the first 12 hours at the NICU, sample of 172 newborns; SGA: small for gestational age.

**Table 4:** Result of the final model of hierarchical multiple logistic regression of determinants associated with death that occurred in the Neonatal Intensive Care Unit.

	Adjusted OR^a^	95%CI	p-value^a^
Birth weight: 750–999 g	4.15	1.06–16.14	0.040
Nonuse of surfactant^b^	0.15	0.48–0.53	0.003
Sepsis^c^	10.68	2.82–40.48	<0.001
SNAPPE II≥27	5.85	1.90–18.05	0.002

OR: Odds Ratio; 95%CI: 95% confidence interval; SNAPPE II: Score for Neonatal Acute Physiology Perinatal Extension;

a OR adjusted for the variables in group III (proximal level);

b among 172 premature newborns who had respiratory distress syndrome, 89 did not use surfactant;

c sample comprised of 246 newborns, 1 unknown.

Note: Hosmer-Lemeshow chi-square = 4.558; p=0.472; Nagelkerke's R^2^=0.713.

## DISCUSSION

In this study, SNAPPE II proved to be a good predictor of death in the NICU. An increase in mortality directly proportional to the SNAPPE II score was verified. The SNAPPE II mortality cutoff point was 27, and each point added to the score increased the odds of dying by 10%. In the cohort study conducted by the Brazilian Neonatal Research Network (*Rede Brasileira de Pesquisas Neonatais* – RBPN), the cutoff point chosen for SNAPPE II as a risk for mortality was >39, taking into account the literature on the subject. In such study, the population consisted of preterm infants (23–33 weeks) weighing between 400 and 1500 g. Conversely, the present study was conducted in a single reference center, including newborns regardless of birth weight and gestational age, and calculated the SNAPPE II cutoff point for the studied population. Both studies used a severity score to predict death.^13^

Among the 247 neonates studied, 35.2% presented SNAPPE II≥27 and 64.8%, <27, with death as the outcome in 61 and 6.2%, respectively. The findings prove the usefulness of SNAPPE II as a predictor of death and the need for improving the care provided to newborns with SNAPPE II≥27, by implementing the protocols and the adherence of professionals, aiming at reducing mortality.

An observational study used the SNAPPE II with 248 newborns, finding an increase in mortality in the score between 41 and 50, cutoff point for death of 37, with sensitivity of 76.9%, specificity of 87.1%, AUC of 0.84 (95%CI 0.79–0.97), and mean score among deaths of 45±19.^14^ A research performed with 288 newborns observed a SNAPPE II cutoff point for mortality of 12.5, with sensitivity of 71%, specificity of 75%, AUC of 0.77 (95%CI 0.69–0.86), and mean among deaths of 21±15.^5^ Institutional differences are verified in the SNAPPE II cutoff points for mortality, reinforcing the recommendation of the authors of the score concerning the individualized calculation of the cutoff point for each institution.

No statistical significance was observed between the SNAPPE II score ranges and the period of occurrence of deaths. Factors other than severity in the first 12 hours of admission may exert influence, as demonstrated in the final logistic regression, with an association between death and sepsis. A systematic review on the period of occurrence and causes of deaths in developing countries found that 62% of deaths occurred in the first three days, and 2/3 in the first 24 hours of life, with almost half related to sepsis.^15^ There was a predominance of deaths in the neonatal period – which suggests its close relationship with the provision of care during pregnancy, childbirth, and to the newborn –, which persist despite advances in perinatal care, evidencing the need for improving the quality of care in all stages.^16^

In the proposed hierarchical framework, the concept of sufficient causes was employed, which considers the chronological order of events based on the hierarchization between levels and the relationship between causal factors, which in turn enables the identification of the causal chain in which interventions can be carried out.^7^ The model showed an association of death with variables of the proximal level: sepsis, SNAPPE II, birth weight, and use of surfactant.

A multicenter cohort study conducted in Northeast Brazil between July and December 2007, involving 627 newborns with gestational age between 23–32^6/7^ weeks and birth weight between 500 and 1499 g, observed 29% of deaths in the early neonatal period (33% within the first 24 hours), with weight <1000 g being one of the variables associated with death <24 hours (OR 2.94; 95%CI 1.32–6.55). The present study was carried out in one of the centers included in the aforementioned cohort study and found 58.8% of deaths in the early neonatal period (18.9% <24 hours). Death was observed as an outcome in 87.2% of neonates weighing <1000 g; moreover, weighing between 750 and 999 g was one of the variables associated with death (OR 4.15; 95%CI 1.06–16.1; p<0.040), demonstrating the need for improving care in this group. The comparison between studies is not possible, considering that their populations are different and the mortality in that specific center during the cohort period is unknown.^17^

In the bivariate analysis, the relationship of deaths with lower gestational age and birth weight is noteworthy. Even though gestational age did not remain in the final logistic regression, it was associated with death, considering that a directly proportional correlation was observed between gestational age and birth weight. Regarding SNAPPE II, an inverse correlation with birth weight and gestational age was verified: the lower the weight and gestational age, the greater the severity, the higher the SNAPPE II score, and the higher the risk of death. A prospective cohort study conducted in Porto Alegre (state of Rio Grande do Sul, Brazil) on newborns with less than 30 weeks and/or birth weight below 1500 g observed mortality inversely proportional to gestational age and birth weight.^18^ A case-control study performed in Chile found an association between death and prematurity (OR 3.1; 95%CI 1.1–8.7; p=0.02) and between death and newborns small for gestational age (OR 4.6; 95%CI 1.7–12.1; p=0.002).^19^

The use of antenatal corticosteroids in 78.6% of the sample reduced the risk and severity of RDS. Among the 172 neonates with the syndrome, 51.7% did not receive surfactant, of which 7.9% died. Not receiving surfactant consisted in a protective effect in the bivariate analysis, which persisted in the multiple logistic regression, a fact that is explained by the greater clinical stability and the absence of medication prescribed for these patients. A multicenter hospital-based study, conducted on 3,623 newborns in 34 NICUs, also found a protective effect concerning the nonuse of surfactant in <1500 g and death (OR 0.54; 95% CI 0.43–0.69; p <0.0001).^20^

A strong association between sepsis and death was observed, and sepsis was the variable with the highest OR in the multiple analysis. Despite technological advances in neonatal care, sepsis remains associated with death in the NICU. It is necessary to develop strategies with preventive measures concerning infection and the adoption of a specific protocol for its approach. According to the World Health Organization (WHO), sepsis is responsible for one million deaths of newborns per year.^21^

A review on early and late sepsis in newborns, carried out in 2016 by authors from Università degli Studi di Bari Aldo Moro, in Italy, found an association of early sepsis with maternal factors – such as premature labor, preterm premature rupture of the membranes >18 hours, and maternal infection – and neonatal factors – such as prematurity, low weight, male sex, and changes in the immune response. Late sepsis had an incidence inversely proportional to birth weight and gestational age, associated with length of hospital stay, parenteral nutrition, central venous catheter, and mechanical ventilation. According to the Italian study, mortality observed in early sepsis was approximately 3% between full-term neonates and 16% between infants <1500 g and, in late sepsis, 36% between infants <1500 g at 8–14 days and 52%, at 15–28 days life.^22^

A review on neonatal sepsis performed by authors from Universidade Federal do Rio Grande do Sul, in 2012, found a high incidence, especially in premature infants, with significant morbidity and mortality, and specific protocols for its prevention and treatment improve the prognosis .^23^ A multicenter study conducted by the RBPN between 2009 and 2010 showed 50% of late sepsis in premature infants <1500 g ranging from 29 to 72% between centers, with 27% accounting for confirmed late sepsis and 23%, clinical sepsis.^24^ A RBPN cohort study on premature infants <1500 g, conducted between 2006 and 2008, also showed that late sepsis is common in premature infants <1500 g, with an incidence of 24% for sepsis confirmed with blood culture, and is associated with higher mortality.^25^ A cohort study performed in Spain between 2006 and 2012 on newborns <30 weeks and/or <1500 g verified a 17.2% incidence of sepsis.^26^ In the present study, a 9.8% incidence of sepsis confirmed with blood culture was verified, although without classification as for the period of occurrence and in a population with different characteristics from the aforementioned studies. A strong association between sepsis and death was observed in the final model (OR 10.68; 95%CI 2.82–40.48; p<0.001).

The use of SNAPPE II in NICUs consists in an important tool for the comprehensive care in perinatal care; allows acquiring knowledge of the severity profile of neonates and the cutoff point for mortality; enables the comparison of mortality rate between services; and subsidizes the provision of quality care to newborns through the implementation of good practices, thus contributing to the reduction of hospital infant mortality and enabling the improvement of child health indicators in Brazil. The limitations of the study were the loss of 92 (24%) of those admitted to the NICU due to transfers to other hospitals, with no knowledge of the final outcome, and the low prevalence of some morbidities, causing difficulties in statistical calculations, with large OR and 95%CI. However, considering that the general objective of the research was to analyze SNAPPE II as a predictor of death in the NICU, these limitations did not harm the results.

In conclusion, SNAPPE II proved to be a good predictor of death in the NICU and mortality was directly proportional to the score range. The cutoff point for the mortality score was 27. Based on such value, the odds of dying in the final stage of multiple regression increases by almost six times; however, no association between SNAPPE II and the period of death was observed. According to the proposed conceptual model, the final analysis with multiple regression showed an association of death with variables of the proximal level: sepsis, SNAPPE II, birth weight between 750 and 999 g, and nonuse of surfactant.

SNAPPE II should be used as an institutionalized management practice, which, together with other initiatives, would enable a better provision of care to newborns at the NICU. In addition, specific protocols for scoring, considering the cutoff point for mortality, and standardized measures based on evidence should be established with the involvement of the healthcare team, aiming at improving the care provided to newborns and reducing infant mortality in hospitals.
